# Analysis of Midterm Readmissions and Related Costs after Open and Endovascular Procedures for Aorto-Iliac Occlusive Disease

**DOI:** 10.3390/life14070798

**Published:** 2024-06-25

**Authors:** Elda Chiara Colacchio, Sabrina Menara, Francesco Squizzato, Michele Piazza, Mirko Menegolo, Franco Grego, Michele Antonello

**Affiliations:** Department of Cardiac, Thoracic, Vascular Sciences and Public Health, Vascular and Endovascular Surgery Section, University of Padova, Azienda Ospedale-Università di Padova, 35128 Padova, Italy

**Keywords:** aorto-iliac occlusive disease, in-hospital readmissions, cost-analysis

## Abstract

*Background.* Readmissions rates and costs were analysed over follow-up for patients who underwent open or endovascular procedures for aorto-iliac occlusive disease (AIOD). *Methods.* Patients who underwent aorto-bifemoral bypass (ABF) or covered kissing stent (CKS) for AIOD from May 2008 to February 2018 were compared in terms of readmission rates, related costs expressed in EUR, freedom from generic readmission (FFGR), and freedom from readmission for surgical reasons (FFRS). *Results.* ABF had a readmission rate of 16% and CKS of 18% (*p* = 0.999). The most common cause of readmission was prosthesis limb or stent occlusion. Time to readmission was longer for ABF (35 months [21–82] vs. 13.5 months [1–68.7] in the CKS group, *p* = 0.334). CKS group had higher cumulative re-hospitalisation, ICU stay, and reintervention costs (11569 ± 2216 SEM, 2405 ± 1125, 5264 ± 1230, respectively) and a trend for more readmissions in the first 36 months, without reaching significance. *Conclusion.* This study reports on a period of time exceeding ninety days. Even if not reaching significance, the CKS group presented a higher trend in readmissions till 36 months and a higher trend in readmission costs, while time-to-readmission was longer in the ABF group.

## 1. Introduction

Peripheral arterial disease (PAD) is a chronic condition, associated with multiple reinterventions and a reported rate of in-hospital readmissions ranging between 15% and 26% [1,2,3,4]. According to Davenport et al., the cumulative readmission rate after vascular procedures performed both for abdominal aortic aneurysms (AAA) and PAD was 17% at 30 days and 27% within 90 days, considering both open and endovascular interventions, with 54% of 30-days readmissions unplanned and related to the index procedure [1]. However, while the 30-days readmission rate in patients with an AAA was 14%, it was 41% for patients who underwent an infrainguinal revascularisation (IIR). At 90 days, one patient had seven readmissions, and fifteen patients had more than one readmission. In fact, patients with PAD have an evolving condition, meaning that if today we treat the aorto-iliac district, tomorrow the same patient could manifest symptoms related to an infrainguinal disease and a novel intervention could be necessary, whether it is planned or unplanned.

Nowadays, aorto-iliac occlusive disease (AIOD) is a pathology treated both by endovascular and open surgical means, even in complex TASC (Trans-Atlantic Inter-Society Consensus Document on Management of Peripheral Arterial Disease) [5,6] C and D cases. In fact, endovascular procedures have shown good results in many reports, even in low-risk patients [7,8], with a 5 years primary patency of 81.4% (vs. 87.3% in the open group, *p* = 0.317) in our experience [7], and a superimposable 6 year primary patency rate in other groups (83%) [8]. The in-hospital costs of these procedures, and their comparison, have been investigated in recent literature [9,10], though without reaching a consensus. In our experience, endovascular procedures had lower costs related to the hospitalisation in the ward and the intensive care unit (ICU) but higher costs related to the implanted materials. In the end, there were no differences in the overall hospitalisation costs between the two procedures, although endovascular procedures showed a higher trend [9]. Conversely, Indes et al. [10] found lower length of stay and costs related to endovascular procedures, even in a multivariate analysis.

We believe that the economic evaluation cannot be complete without the analysis of readmissions over the follow-up period. Of course, hospital readmissions are a major cause of increased costs in the health care system. Davenport et al. have reported a 16.5% 30-days rate of readmissions after open and endovascular PAD aorto-iliac and infrainguinal procedures [1], stating that the rate of readmissions after open interventions was double that of endovascular procedures. Orr et al. [11] found that readmission costs were higher for endovascular procedures, and this result was even more evident in patients with AIOD.

In this article, we reported our experience with readmissions after aorto-bifemoral bypass (ABF) and kissing technique with covered stents (covered kissing stenting—CKS) performed for AIOD, comparing rates and costs of the two techniques over the whole follow-up period.

## 2. Materials and Methods

### 2.1. Patients and Definitions

All patients undergoing revascularization for aorto-iliac occlusive disease from May 2008 to February 2018 were included in a dedicated database. In this retrospective observational cohort study, we investigated readmissions of patients previously included in our in-hospital analysis [9]. The total cohort was divided in two groups according to the index procedure: ABF and CKS. These were the only two procedures included in the cohort. For the CKS group, we included both balloon expandable and self-expandable covered stents. We excluded patients undergoing bilateral iliac revascularisation using other techniques (e.g., covered endovascular reconstruction of the aortic bifurcation—CERAB, or bilateral iliac stenting without a kissing conformation) or using balloon expandable or self-expandable bare metal stents. We also excluded bilateral iliac revascularization with two different techniques (e.g., unilateral iliac stenting and femoro-femoral crossover bypass), unilateral treatments (open or endo), less complex AIOD such as TASC A and B lesions, cases with associated aneurysms and dissections, and patients undergoing another planned intervention, such another district revascularisation, during the same hospitalisation. For included patients, we collected demographics, cardiovascular risk factors, comorbidities, such as the presence of coronary artery disease with or without previous revascularisation, pulmonary diseases, arterial hypertension, dyslipidaemia, smoking habit, diabetes mellitus, and chronic renal insufficiency. We also registered the extent of the disease according to the TASC classification [6], the clinical status according to the Rutherford classification [12], and the grade of frailty of patients according to the American Society of Anaesthesiologists physical status examination (ASA score) and the Society for Vascular Surgery grading scale (SVS score) [13].

We identified both medical and surgical readmissions. Medical readmissions were defined as return to the hospital within 30 days from the index procedure for reasons not related to the intervention itself (e.g., readmissions for cardiac or pulmonary events). Surgical readmissions were defined as return to the hospital for reasons technically related to the index procedure, both requiring or not requiring reintervention. We registered all surgical readmissions for vascular procedures during the whole follow-up period, but only those related to the index procedure were considered in the cost analysis.

For each medical readmission, we registered the cause, the total length of stay (LOS), the LOS in an intensive care unit (ICU), the number and cost of blood transfusions, the cost of the entire LOS, and the cost of the ICU stay.

For surgical readmissions, we registered cause and reintervention type, the length of each procedure in minutes, and the duration of the patient’s stay in the operatory room (OR) in order to calculate the salary of surgeons, anaesthesiologists, nurses, and healthcare workers. Moreover, we registered which material was used for the novel procedure, as well as the costs related to it. Finally, as for medical readmissions, we noted total LOS, ICU stay, blood transfusions, and the costs related to it. Surgical reasons considered as leading to readmission or complicating the hospitalisation were schematized in occlusion-related (i.e., stent or prosthesis occlusion or stenosis, anastomosis stenosis, or stent recoiling), hematoma-related, and infection-related. We considered both unplanned and planned readmissions for surgical procedures.

Costs were further divided in direct and indirect costs (DC; IC), as previously detailed [9], and were reported on the price base of the financial year 2018. The cost of the ward included blood transfusions.

Major adverse events (MAE) included medical complications as acute kidney injury [14], acute myocardial infarction diagnosed with electrocardiographic abnormalities and/or significative troponin values increases, arrhythmias leading to a change in medical therapy and/or electric cardioversion, heart failure, respiratory failure requiring ventilatory support, major stroke, and intestinal ischaemia. 

Follow-up consisted in medical examination and ultrasound, adding computed tomography angiography (CTA) or simple CT where needed. Ultrasound was used to detect changes in arterial flow at femoral arteries level, or anastomosis pseudoaneurysms. Whenever we had a suspicion of intrastent restenosis, stent recoiling, stent or prosthesis occlusion. or pseudoaneurysms, a CTA was requested. Follow-up was always performed 6 months at the latest after the index procedure and yearly afterward. Patients not living in our region were reached by telephone to investigate their health status and whether they had undergone further interventions.

### 2.2. Endpoints

Primary endpoints were the rates of overall, medical, and surgical readmissions, each compared between the two groups (ABF and CKS). We subsequently analysed costs related to surgical readmissions, focusing on ward, ICU, and reintervention itself, and compared them between the two groups.

Secondary endpoints were freedom from generic readmission (FFGR), that is both for surgical and medical reasons, and freedom from readmissions for surgical reasons (FFRS).

### 2.3. Statistical Analysis

Analysis were performed using R 4.1.0 (R Foundation for Statistical Computing. Vienna, Austria) and Prism GraphPad 10.0.0 (GraphPad Inc., San Diego, CA, USA). We reported patients’ baseline characteristics as mean ± standard deviation (SD) or median + interquartile range (IQR), and frequency + percentage. Unpaired *t*-tests or Wilcoxon rank sum test were used for continuous variables, as appropriate. Chi-Squared or Fisher’s exact test were used for categorical variables.

Kaplan–Meier survival curves were estimated for FFGR and FFRS, comparing curves with the log-rank test, only admitting a standard error < 10%.

Statistical significance was considered with a two-tailed *p*-value < 0.05.

## 3. Results

Of the 107 patients who underwent aorto-iliac revascularisation in our vascular unit from May 2008 to February 2018, 100 were previously analysed for the in-hospital analysis [9] and were subsequently selected for the present study. Mean age was 67 ± 9 years and 71% of patients were males. Patients’ demographics and principal comorbidities were analysed in our previous paper [9] and are reported in Table 1. Median follow-up was 41 months (range 1–176), and 21 patients (21%) were lost to follow-up.

Readmission rates are detailed in Table 2: no differences were found between the two groups in terms of readmission rates and reinterventions. Five patients (5%) experienced at least one readmission in the first 30 post-operative days, two in the ABF group, and three in the CKS group. In the ABF group, one patient had a groin hematoma that was treated conservatively, and another had acute heart failure with pneumonia, not requiring a hospitalisation in the ICU. In the CKS group, one patient had urinary tract infection requiring intravenous antibiotic therapy, another patient underwent a novel procedure with the positioning of a covered stent in a ruptured common iliac artery, and the third patient had a bilateral acute stent occlusion and underwent aorto-iliac endarterectomy. In the CKS group, four more patients underwent a novel intervention during follow-up, including two femoral endarterectomies, one femoro-posterior tibial bypass, and one percutaneous transluminal angioplasty (PTA) of the superficial femoral artery (SFA), yet all these procedures were performed in a district other than the one treated in the index procedure; as such, we did not include these reinterventions in the cost-analysis.

Concerning readmissions > 30 days with reinterventions in the ABF group, two patients experienced a severe stenosis of one distal ABF anastomosis and underwent a femoral endarterectomy and a prosthetic-profunda femoris artery bypass; one patient had a severe stenosis of a graft limb, which was treated by stenting; three patients had a limb graft occlusion: two underwent femoral endarterectomy and thrombo-embolectomy and one underwent thrombo-embolectomy and a prosthetic-profunda femoris artery bypass. This second patient had two more readmissions for the same reason, and underwent thrombo-embolectomy in both cases, with a subsequent prosthetic-popliteal bypass. Finally, another patient had a second readmission with a LOS of 19 days because of a suspicion of infection of the graft located at the left groin and occlusion of the common and profunda femoral arteries, along with pre-existent occlusion of the superficial femoral artery; we performed a thrombo-embolectomy of the graft and the common and profunda femoral arteries and a femoro-popliteal bypass using heparin-bonded expanded polytetrafluoroethylene (ePTFE) graft. The microbiological analysis of the intraoperative samples excluded infection of the previous graft, although the patient underwent intravenous antibiotic therapy until we had the results of the analysis.

Reinterventions during readmissions > 30 days in the CKS group were performed for stent occlusion and included two ABF and three thrombo-embolectomy with a Fogarty catheter and novel iliac stenting. In one case, the patient had rest pain following stent occlusion and was treated conservatively with pentoxifylline with complete recovery from symptoms.

Reasons of readmissions for surgical reasons are synthetized in Table 3.

Considering the subgroup of patients undergoing readmission for a surgical reason (Table 4), median time to readmission was higher for patients in the ABF group, yet not significantly (Table 4), while more patients in the CKS group needed ICU. Concerning costs, our analysis showed a higher trend in patients belonging to the CKS group, although also not reaching significance (*p* = 0.139, *p* = 0.126, *p* = 0.561, *p* = 0.387 for ward, ICU, intervention, and cumulative hospitalisation cost, respectively).

**Figure 1:**
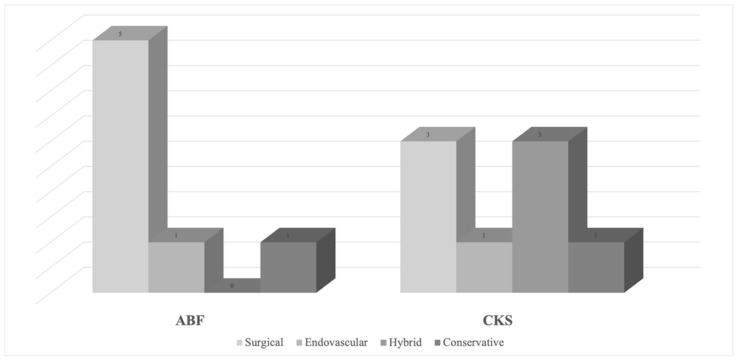
Schematic details of reintervention types on both groups. ABF: aorto-bifemoral bypass; CKS: covered kissing stent.

In the ABF group, most patients (5/7, 71.4%) underwent open surgical repair; in the CKS group, three patients underwent open surgical repair, three patients a hybrid approach, and only one patient was treated by exclusive endovascular means (Figure 1). 

**Figure 2:**
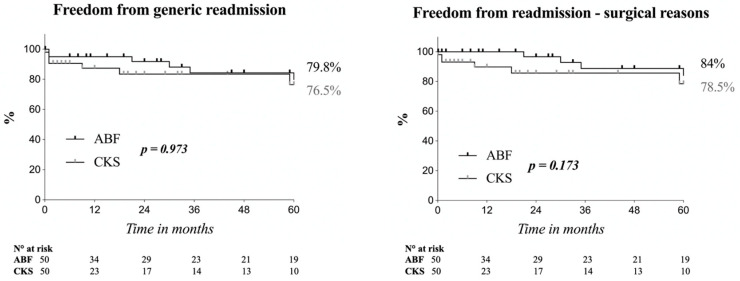
Kaplan–Meier estimates on freedom from generic readmission (FFGR) and freedom from readmission for surgical reasons (FFRS) in both groups, based on a time-to-event analysis. The log-rank test did not show any differences for both analyses, although the CKS group did have a higher rate of readmissions both generic and for surgical reasons in the first 36 months. Standard error < 10%. ABF: aorto-bifemoral bypass; CKS: covered kissing stent.

Finally, survival analysis did not point out significant differences in freedom from readmissions, although the CKS group did have a lower rate of FFRS (78.5% vs. 84% in the ABF group, *p* = 0.173) in the first 36 months, with a subsequent overlapping of the curves (Figure 2).

## 4. Discussion

### 4.1. General Considerations

Patients affected by vascular diseases present a higher degree of frailty, often being affected by other comorbidities such as diabetes mellitus, cardiac diseases, arterial hypertension, chronic renal insufficiency, and smoking habit, with a related higher risk of complications in the postoperative period.

Patients affected by PAD usually present a higher number of comorbidities, with associated longer LOS and augmented need for ICU stay during the hospitalisation for revascularisation procedures. Over the years, literature has begun to focus on the economic side of common PAD revascularisation interventions, yet most papers only analyse the hospitalisation for the first procedure. Doshi et al. [15] compared bypass surgery versus endovascular procedures for lower extremity PAD, finding a lower mortality, a lower LOS, and lower costs of hospitalization for patients undergoing endovascular procedures, yet not distinguishing interventions by anatomic district. Indes et al. [10] reported their experience with 4119 patients, more than half treated with open repair, focusing on AIOD. Their analysis showed that patients treated by endovascular means presented a higher comorbidity score; however, they had lower LOS, lower in-hospital complication rate, and lower overall hospitalisation costs. Our previous paper [9] on AIOD revascularisation compared two types of interventions: ABF bypass and CKS. Unlike other authors [10,15], the comparison of overall costs of hospitalisation for the two groups did not show statistical significance, yet there was a higher trend for endovascular procedures, related to the higher costs of implanted material (i.e., stents). Although the grade of frailty of the endovascular cohort was higher, with a SVS score of 0.97 ± 0.46 for the endovascular cohort vs. 0.81 ± 0.46 for the open surgery cohort (*p* = 0.087), the endovascular group had a lower need for ICU stay and a lower LOS, and this reduced the overall hospitalisation costs.

However, to obtain a complete picture of PAD, we believe it is mandatory to consider readmissions.

### 4.2. Previous Articles on Readmissions

Some articles on readmissions and related costs are reported in the literature, but they usually analyse all vascular intervention types and for a period not exceeding ninety days.

Orr et al. [11] reported on readmissions in 219 cases of the most common vascular procedures, with a readmission rate of 17% in the first 30 post-operative days and of 29% within 90 days, comparing results between open and endovascular interventions. The overall median readmission cost was USD 10,700. Most readmissions were related to wound complications, and approximately half of them (55%) required a novel intervention. To our best knowledge, this is the only report also analyzing the economic side of the principal vascular interventions, and costs were compared between endovascular and open surgical groups for three subgroups of patients: abdominal aortic aneurysms, aorto-iliac disease, and infrainguinal disease. For the aorto-iliac disease, readmission costs were higher in the endovascular group; however, total cumulative costs (index procedure and readmission) were higher in the open surgical group, without statistical significance (*p* = 0.15).

According to Duwayri et al. [16], the most common cause for readmission was wound complications (36%) followed by “procedure specific” complications (22%), with a 30-days readmission rate of 9.6%. The authors did not specifically focus on AIOD or even PAD interventions, and analyzed a wide spectrum of procedures, from carotid revascularizations to AAA procedures to PAD revascularizations. However, they did stratify the readmission rate by procedure type, and it was clear that the rate of re-hospitalization after infrainguinal revascularization procedures was the highest (15.6%), followed by supra-inguinal procedures (13.5%), and the combined rate of readmissions after infrainguinal and supra-inguinal procedures was 40%. While overall costs of readmissions were calculated for each subgroup of patients (e.g., carotid artery stenting, open AAA repair), the author did not compare costs for open and endovascular readmissions after PAD procedures.

Davenport et al. [1] were the only authors that focused also on readmissions related to the aorto-iliac and infrainguinal disease, identifying the use of blood transfusions, body mass index between 30 and 35, and operative time > 260 min as significant independent factors associated with readmissions within 30 days.

According to Vogel et al. [4], no significant differences were shown between open and endovascular lower extremities revascularizations concerning readmission rates in patients suffering from intermittent claudication, rest pain, and ulcers or gangrene. Moreover, a more severe clinical status (gangrene) was associated with a higher readmission rate in both groups (21.1% in the open group vs. 19.5% in the endovascular one), but endovascular procedures were not associated with a lower readmission rate. However, the authors did not specify if the interventions concerned aorto-iliac or infrainguinal district.

### 4.3. Our Experience and Comparison to Other Reports

The aim of our work was to compare open and endovascular procedures performed for AIOD in the follow-up period, analyzing costs of readmissions. To our best knowledge, this is the first article comparing readmissions after open and endovascular procedures exclusively focusing on AIOD revascularizations and related costs and extending the analysis period to more than 90 days. Our cohort of patients was previously studied for in-hospital cost analysis [9]. 

First, we did not have planned readmissions. Unlike Davenport et al. [1], we did not find significant differences in readmission rates between the two groups, both in the period within and exceeding 30 post-operative days, and in reintervention rates. Again, differently from previously cited literature [1,11,16], the most frequent etiology for readmission was occlusion of the prosthetic limb (42.8%) or stent (75%)for the ABF and CKS group, respectively, followed by femoral anastomosis stenosis (28.6%) and stent recoiling (12.5%), while one patient in each group suffered from an hemorrhagic complication, requiring reintervention only in the CKS group (iliac rupture). One patient in the ABF was suspected of prosthetic infection, while other wound complications (i.e., dehiscence) did not represent an etiology for readmission because they involved only the superficial plans of the wound. Davenport et al. found that the most frequent cause for readmission was surgical site infection (35.8%) followed by graft complications, not further detailed, other infections and sepsis, and wound complications [1], yet these rates were not specific for the aorto-iliac district. Orr et al. also found that the surgical site infection was the main cause for readmissions, again without specifically inquiring on the aorto-iliac district [11]. Without any stratification by anatomic arterial district, in unadjusted analysis, Vogel et al. [4] found a non-significant slightly higher rate in readmission rated for endovascular procedures performed for rest pain (18.2% vs. 14% of open procedures, *p* = 0.43) and claudication (11.3% vs. 10.2%, *p* = 0.69). Despite this, in the multivariable analysis with adjustment for race, sex, length of stay, comorbidities, and laboratory values outside the normal range, the endovascular treatment did not represent a risk factor for readmission [4]. 

Interestingly, even if readmission rates did not differ between the two groups, in our report the time-to-readmission was longer in the ABF group (35 [21–82] months vs. 13.5 [1–68.7] months for CKS, *p* = 0.334), and this was confirmed by survival analysis. Even if no significant differences were eventually found in FFGR and FFRS, the CKS curve showed a trend for higher readmission rate, with the curves’ gap more pronounced between 12 and 36 months of follow-up.

Six and seven patients required a reintervention in the ABF and CKS group, respectively. Reinterventions in the ABF group often required an open surgical procedure (5/6 patients, 83.3%), while in the CKS group, three patients underwent an open reintervention, three patients underwent a hybrid reintervention, and only one patient required an exclusive endovascular approach. This may explain why patients in the CKS group were associated with higher ward and ICU costs during readmissions. In fact, our cohort was subjected to the index procedure between 2008 and 2018, when an endovascular approach was usually proposed to patients with a more complex medical history. Thus, it is understandable why these patients, when undergoing a more invasive intervention, required ICU admission and were associated with higher in-hospital costs. Nowadays, given the good results of these procedures, an endovascular-first approach is often offered to fit patients; as a result, costs associated with ICU and ward admissions could be different when analysing a more contemporary group of patients. 

Focusing on the economic side of the analysis, in our experience, even though the CKS group showed a trend to higher overall readmission costs and reintervention costs, no significant differences were found between the two groups. We think that the costs of the ward and ICU admission could be explained because of the high frailty of these patients, while the higher costs of the reintervention are probably related to the endovascular material used during procedures. In fact, in the endovascular cohort, four patients underwent hybrid procedures involving stent deployment. 

The absence of difference in costs between the ABF and CKS cohorts is partially in line with Orr et al. [11]. Performing an analysis of the first 90 post-operative days after aorto-iliac revascularisations, the authors found that the costs of readmissions after endovascular procedures exceeded of 26% the cost of the hospitalisation for the index procedure. We believe that the different trend between this analysis and ours could be explained because of the length of the analysed period. In fact, our analysis showed that in both the FFGR and FFRS survival curves, the gap was more evident between 12 and 36 months, which is after the end of the period observed by Orr et al.

### 4.4. Limitations

The most important limitation of this analysis is the low number of patients and events. In fact, some results had a higher trend in one group or the other without reaching statistical significance, and this may be due to a power problem. Moreover, this is a retrospective single-centre study, including a period of 10 years, with a progressive change in procedure techniques and indications to intervention. In fact, the type of intervention for open revascularisations has not undergone significant changes over the examined period, except for the modification of the type of prosthesis in some cases (i.e., use of antimicrobial material). Conversely, endovascular procedures are constantly evolving, for example regarding the type of femoral access that went from surgical to percutaneous, adding devices employed during the intervention. Finally, the last limitation is the adherence to follow-up that was not complete (21% lost), which unfortunately is a common problem in vascular surgery studies.

## 5. Conclusions

Time-to-readmission was longer for patients in the ABF group, but no significant difference in readmission rates was found between the two groups, both before and after thirty post-operative days. Our analysis focused on a period of time exceeding ninety days and showed a higher trend in readmissions in the CKS group till thirty-six months, with a progressive overlapping of freedom from readmission curves with a longer follow-up. CKS group showed a higher trend in surgical readmission costs, including ward, ICU, and reintervention, with a larger need of ICU stay. To validate our findings, future reports with a higher number of patients are needed.

## Figures and Tables

**Table 1:** Patients’ demographics and main comorbidities.

	ABF (*n* = 50)	CKS (*n* = 50)	*p* Values
Age, *n*° (%)	66.6 ± 7.8	68.3 ± 9.8	0.328
Male sex, *n*° (%)	36 (72)	35 (70)	0.825
Diabetes mellitus, *n*° (%)	19 (38)	12 (24)	0.269
Arterial hypertension, *n*° (%)	43 (86)	44 (88)	0.766
Dyslipidaemia, *n*° (%)	40 (80)	26 (52)	0.698
Renal insufficiency, *n*° (%)	8 (16)	6 (12)	0.774
Coronary artery disease, *n*° (%)	20 (40)	22 (44)	0.685
SVS total score	0.81 ± 0.46	0.97 ± 0.46	0.087

**Table 2:** Readmission rate during follow-up.

	ABF (*n* = 50)	CKS (*n* = 50)	*p* Values
Cumulative < 30 days readmissions, *n*° (%)	2 (4)	3 (6)	>0.999
Medical < 30 days readmissions, *n*° (%)	1 (2)	1 (2)	>0.999
Surgical < 30 days readmissions ^a^, *n*° (%)	1 (2)	2 (4)	>0.999
Cumulative readmissions > 30 days during follow-up ^a^, *n*° (%)	6 (12)	6 (12)	>0.999
Cumulative readmissions during follow-up, *n*° (%)	8 (16)	9 (18)	>0.999
Patients undergoing reintervention during follow-up, *n*° (%)	6 (12)	7 (14)	>0.999

^a^ for reasons linked to the index surgical procedure, but not necessarily undergoing a novel intervention.

**Table 3:** Synthesis of readmission for surgical reasons.

ABF (*n* = 7/50)	*n*° (%)	CKS (*n* = 8/50)	*n*° (%)
Femoral anastomosis stenosis Limb occlusion Groin hematoma Suspected prosthesis infection	2 (28.6) 3 (42.8) 1 (14.3) 1 (14.3)	Stent occlusion Stent recoiling Iliac rupture	6 (75) 1 (12.5) 1 (12.5)

**Table 4:** Details on cumulative readmissions (both < 30 days and >30 days) for surgical reasons over follow-up. Costs are expressed in euros.

	ABF (*n* = 7/50)	CKS (*n* = 8/50)	*p* Values
Time to readmission in months, median [IQR]	35 [21–82]	13.5 [1–68.7]	0.334
Length of stay, median [IQR]	8 [3–10]	7.5 [6.2–17.7]	0.554
Readmissions requiring ICU stay, *n*° (%)	2 (28)	5 (62.5)	0.592
Readmissions requiring reintervention, *n*° (%)	6 (85.7)	7 (87.5)	>0.999
Cost of the ward ^b^, mean ± SEM	2328 ± 447.3	3900 ± 843.6	0.139
Cost of the ICU, mean ± SEM	392.7 ± 253.5	2405 ± 1125	0.126
Cost of intervention, mean ± SEM	4188 ± 1320	5264 ± 1230	0.561
Cumulative cost of the hospitalisation, mean ± SEM	8539 ± 2591	11569 ± 2216	0.387

^b^ comprehensive of blood transfusions.

## Data Availability

The majority of data are already in the “Results” section. Further data are available on request from the corresponding author.
